# The site of water stress governs the pattern of ABA synthesis and transport in peanut

**DOI:** 10.1038/srep32143

**Published:** 2016-10-03

**Authors:** Bo Hu, Jiajia Cao, Kui Ge, Ling Li

**Affiliations:** 1 Guangdong Provincial Key Lab of Biotechnology for Plant Development, College of Life Sciences, South China Normal University, Guangzhou, 510631, P. R. China

## Abstract

Abscisic acid (ABA) is one of the most important phytohormones involved in stress responses in plants. However, knowledge of the effect on ABA distribution and transport of water stress at different sites on the plant is limited. In this study, water stress imposed on peanut leaves or roots by treatment with PEG 6000 is termed “leaf stress” or “root stress”, respectively. Immunoenzyme localization technolony was first used to detect ABA distribution in peanut. Under root stress, ABA biosynthesis and distribution level were all more pronounced in root than in leaf. However, ABA transport and the ability to induce stomatal closure were still better in leaf than in root during root stress; However, ABA biosynthesis initially increased in leaf, then rapidly accumulated in the vascular cambium of leaves and induced stomatal closure under leaf stress; ABA produced in root tissues was also transported to leaf tissues to maintain stomatal closure. The vascular system was involved in the coordination and integration of this complex regulatory mechanism for ABA signal accumulation. Water stress subject to root or leaf results in different of ABA biosynthesis and transport ability that trigger stoma close in peanut.

In plants, abscisic acid (ABA) plays a very important role in mediating host responses to both biotic and abiotic stresses^1^,^2^. ABA modulates physiological changes at the cellular level, resulting in both response and adaptation to abiotic stresses. Thus, it regulates gene expression and stomatal closure, thereby preventing water loss, and protects cells against the damaging effects of water stress^3^.

An understanding of the ABA accumulation pattern within the root system is essential to predict long-distance ABA signaling responses to soil drying^4^. Although ABA biosynthesis and metabolism occurs predominantly in vascular tissues, ABA has functions in all tissues, from roots to leaves, suggesting that it is transported throughout the plant^5^. Stomatal closure occurs in leaves even when only the roots experience drought stress^6^, indicating that signals generated in the roots are able to affect a response in the leaves. It has also been reported that ABA concentrations in the xylem sap correlate with stomatal conductance, while bulk leaf ABA concentrations remain constant^7^. These findings suggest that ABA synthesized in root tissues is transported to the guard cells via the xylem. On the other hand, stomatal closure can occur even in the absence of root-derived ABA. Reciprocal grafting between ABA-deficient mutants and wild-type plants in tomato and Arabidopsis demonstrated that stomatal closure is affected by the leaf (shoot) genotype, not the root genotype^8^. However, increased ABA levels are not observed if roots are exposed to water stress without changing the water status in leaves, indicating that leaves are the main sites of ABA biosynthesis during water stress^9^. ABA levels increase both in leaves (shoots) and roots when intact whole seedlings are exposed to water stress, whereas ABA accumulates mostly in shoots when detached shoots and roots are separately water-stressed^10^. Further understanding of the factors causing these different responses to different sites of water stress is essential for modeling ABA biosynthesis and transport in response to drying.

The peanut plant (*Arachis hypogaea* L.) is the fourth most important cultivated source of edible oil and protein in the world^11^. Drought is one of the major abiotic stresses that limit the growth and production of peanuts^12^. In our previous study in peanut, we found that ABA was predominantly distributed in the leaf or root at various developmental stages^12^, but it is currently unknown how water stress at different sites of the plant affects ABA biosynthesis and transport. Biochemical and genetic evidence shows that the cleavage of 9-cis-epoxycarotenoids, which is catalyzed by 9-cis-epoxycarotenoid dioxygenase (NCED), is the rate-limiting step in the ABA biosynthetic pathway^13^. AhNCED1 (*A. hypogaea* 9-cis epoxycarotenoid dioxygenase 1) has been cloned from peanut (GenBank accession no. AJ574819) and immunostaining has been used to show that both AhNCED1 and ABA levels increase rapidly in the vascular parenchyma of plants subjected to water stress; AhNCED1 distribution reflects that of ABA^14^. These results provide insights into AhNCED1-mediated ABA biosynthesis and distribution in peanut, and its importance for a rapid response to water stress. We previously suggested that the regional distribution patterns of ABA biosynthesis in seedling-stage peanut plants in response to water stress were root-stem-leaf^12^. In fruiting-stage plants, however, the distribution pattern of ABA was first in leaf, then in stem, and last in root. And then we wanted to investigate whether water stress at different sites could influence stomatal closure in peanut. This study therefore aimed to assess how ABA biosynthesis and transport, and their influence on stomatal closure, depend on the site of imposition of water stress in peanut.

## Results

### Leaf ABA syntheses is triggered at different times during root stress and leaf stress

**Figure 1:**
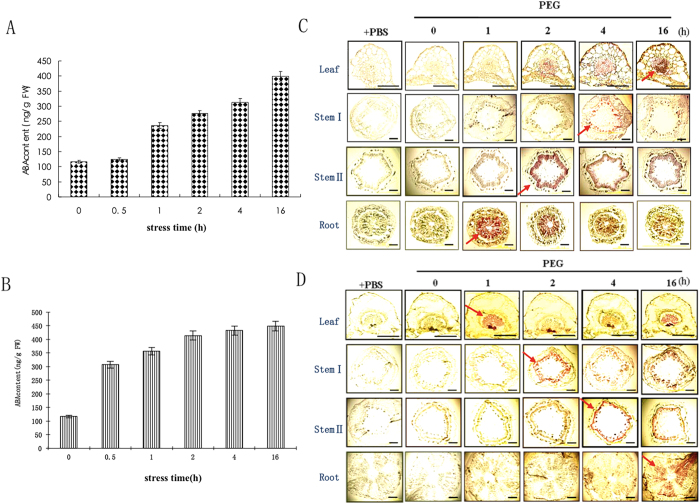
Effect of root and leaf stress on ABA level in leaves and AhNCED1 distribution in peanut tissues. Plants were treated with PEG 6000 (30%) for the times shown. (**A**) Leaf ABA content under root stress. Data are representative of a single in three independent experiments. (**B**) Leaf ABA content under leaf stress. Data are representative of a single in three independent experiments. (**C**) Immunostaining of AhNCED1 in peanut under root stress. An arrow indicates the earliest time point at which AhNCED1 was detected in each tissue. (**D**) Immunostaining of AhNCED1 in peanut under leaf stress. An arrow indicates the earliest time point at which AhNCED1 was detected in each tissue. Controls were conducted with PBS, omitting the primary antibodies. The images shown are representative of three independent experiments with sections from three plant tissues. Bar = 50 μm for the leaf panels, and 100 μm for the stem and root panels.

Leaf ABA content was initially low, but then gradually increased during both root and leaf stress treatments (Fig. 1A,B). ABA levels increased more rapidly in leaves following the imposition of leaf stress, however. Immunostaining showed that AhNCED1 (the rate-limiting enzyme in ABA biosynthesis) was induced in the root vascular parenchyma after root stress for 1 h. The enzyme was detected somewhat later in stem II and stem I, at 2 and 4 h, respectively, but AhNCED1 was not detected in the leaf vascular parenchyma until 16 h (Fig. 1C). Interestingly, the inverse pattern was observed during leaf stress. Thus, AhNCED1 was detected in the vascular parenchyma of leaves after only 1 h of leaf stress (Fig. 1D) and in stem I and stem II after 2 and 4 h, respectively. Notably, no significant increase in AhNCED1 immunostaining signal was observed in root vascular parenchyma until 16 h after leaf stress was imposed (Fig. 1D).

### ABA first synthesized and distributed in root of peanut under root stress

**Figure 2:**
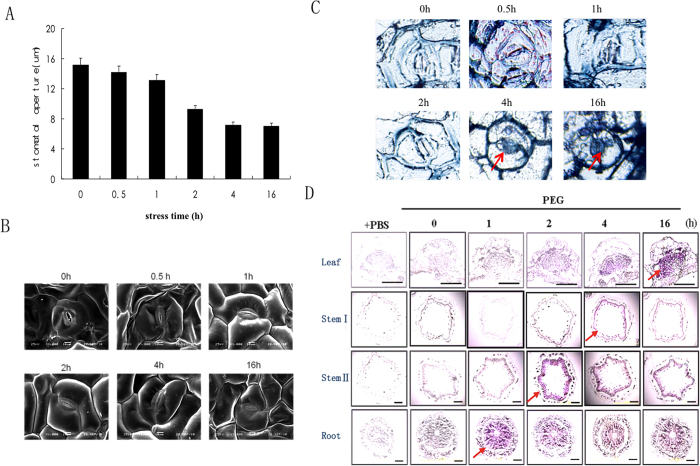
Changes in stomatal aperture and ABA distribution in peanut in response to root stress. Roots were treated with PEG 6000 (30%) for the times shown. (**A**) Change in leaf stomatal aperture over time. Data are representative of 25 stomata in four samples in each of three independent experiments. (**B**) SEM images of stomatal closure during root stress (Bar = 10 μm). (**C**) ABA distribution in stomata. The blue staining in stomata indicates the point at which ABA was detected. (**D**) Immunostaining of ABA in plant tissues, purple staining (arrows) indicates the presence of ABA. An arrow indicates the earliest time point at which ABA was detected in each tissue. Controls were conducted with PBS, omitting the primary antibodies. The images shown are representative of three independent experiments with sections from three plant tissues. Bar = 50 μm for the leaf panel and 100 μm for the stem and root panels.

During root stress, there was relatively little change in stomatal aperture up to 1 h, but then it decreased gradually at 2 h. The stomatal aperture fell by 44% (Fig. 2A,B). ABA was barely detectable by immunostaining in either stomata or organs prior to the imposition of water stress (Fig. 2C,D). However, there was a strong ABA immunostaining signal in stomata after 4 h of root stress, continuing through the 16 h time point (Fig. 2C). In the vascular parenchyma of roots, a strong ABA signal was observed after 1 h (Fig. 2D), while in stem II and stem Ι, ABA was detected at 2 h and 4 h, respectively. However, a strong ABA immunostaining signal was undetectable in the leaf vascular parenchyma until 16 h post-treatment (Fig. 2D). Overall, our data suggest that ABA biosynthesis and distribution are first initiated in root tissue in seedling-stage peanut in response to root stress.

**Figure 3:**
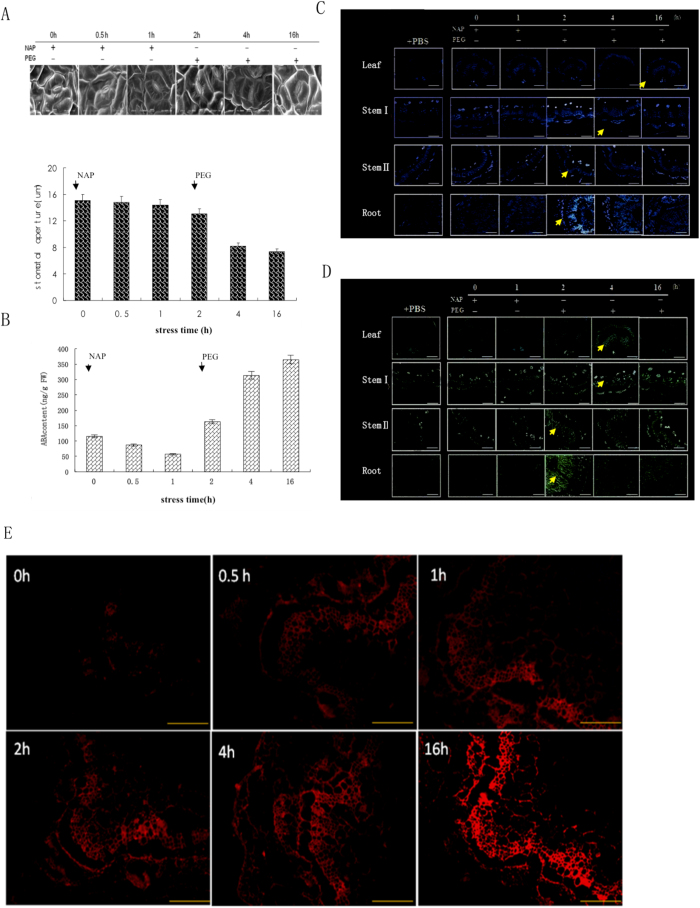
Effect of NAP on stomatal aperture, ABA level, and distribution of ABA and AhNCED1 in peanut in response to root stress. (**A**) Stomatal aperture in leaves on treatment of roots with NAP for 2 h, followed by PEG treatment from 2 h up to 16 h. (**B**) ABA levels in leaves following the same treatment regime described for A. Bars are means (±SD) of n = 3. (**C**) AhNCED1 immunostaining of leaf, stem and root following the same treatment regime described for A. Control samples were treated with PBS; blue fluorescence (arrows) indicates the presence of AhNCED1; no color indicates a lack of staining. (**D**) ABA immunostaining of leaf, stem and root following the same treatment regime described for A. Control samples were treated with PBS; green fluorescence (arrows) indicates the presence of ABA; no color indicates a lack of staining. Bars = 50 μm for leaf panels and 100 μm for stem and root panels. (**E**) ABA distribution at the subcellular level in root of peanut following root stress. Red fluorescence indicates positive immunostaining; no color indicates a lack of staining. Bars = 100 μm.

To confirm this, we studied the effects of naproxen (NAP), a potent ABA biosynthesis inhibitor due to its action on NCED^12^, on stomatal aperture and ABA distribution in different organs. The plants were first treated with NAP for 2 h. This had little apparent effect on stomatal aperture (Fig. 3A), but the ABA content in leaf decreased, falling by 49% (Fig. 3B). When NAP treatment was stopped after 2 h, and water stress treatment started, the ABA content in leaf increased. The mean stomatal aperture rapidly declined, reaching 68% (Fig. 3A).

During NAP treatment, immunostaining of both AhNCED1 and ABA was very weak in all peanut tissues. After 2 h of root stress, strong immunostaining of AhNCED1 was detected in taproot vascular cambium, but the signal diminished at later time points. Similar observations were made for the vascular tissue of stem II, while in the vascular tissue of stem Ι and in leaf vein AhNCED1 signal was first detected at 4 h and at 16 h, respectively (Fig. 3C). For ABA, immunostaining signal was observed at the 2 h time point in the vascular cambium of the taproot, at 2 h in the vascular tissue of stem II, at 4 h in the vascular tissue of stem Ι, and at 4 h in the cambium and xylem of leaf (Fig. 3D).

To determine the location of ABA biosynthesis, we also analyzed ABA distribution at the subcellular level in the vascular tissue of taproot subjected to water stress. ABA immunostaining signals began to appear in taproot vascular cambium at 0.5 h. As the stress continued, ABA levels in the root vascular cambium increased, and diffused to the xylem and epidermal cells and other areas (Fig. 3E). So in response to root stress, ABA is initially synthesized in root where it accumulates before being transported to other tissues including leaf, where it induces stomatal closure.

### Under leaf stress, ABA first synthesized and distributed in leaf and related to induce stomata closed

**Figure 4:**
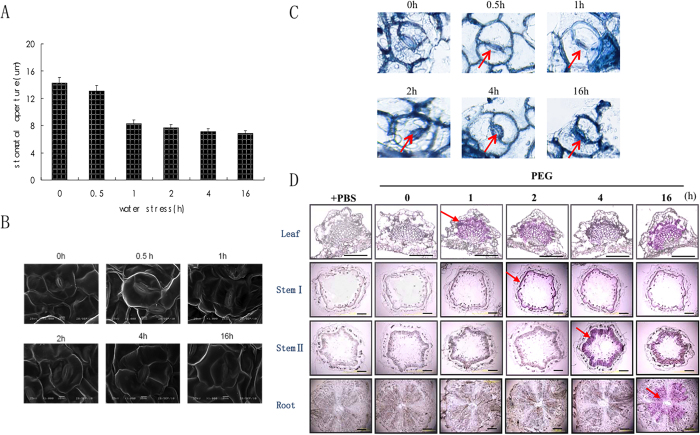
Changes in stomatal aperture and ABA distribution in peanut in response to leaf stress. Plants were treated with PEG 6000 (30%) for the times shown. (**A**) Change in stomatal aperture during leaf stress. Data are representative of four samples in three independent experiments. (**B**) SEM images of stomatal closure during leaf stress (Bar = 10 μm). (**C**) ABA distribution in stomata, blue staining (arrows) indicates the presence of ABA in stomata. (**D**) Immunostaining of ABA, purple staining (arrows) indicates the presence of ABA. An arrow indicates the earliest time point at which ABA was detected in each tissue. Controls were conducted with PBS, omitting the primary antibodies. The images shown are representative of three independent experiments with sections from three plant tissues. Bar = 50 μm for leaf panels and 100 μm for stem and root panels.

In contrast to the slow response to root stress, stomatal aperture began to decrease (by 11%) as early as 0.5 h after the imposition of leaf stress and continued to decrease throughout the experiment (Fig. 4A,B). Similarly, strong positive immunostaining for ABA was observed in stomata by 1 h of water stress (Fig. 4C); elevated ABA levels were also detected in the vascular parenchyma of leaves at the same time (Fig. 4D). In stems or roots, ABA became apparent at later time points, and not until 16 h after treatment in root vascular parenchyma (Fig. 4D). Thus, these results show that ABA is initially synthesized mainly in peanut leaves in response to leaf stress.

**Figure 5:**
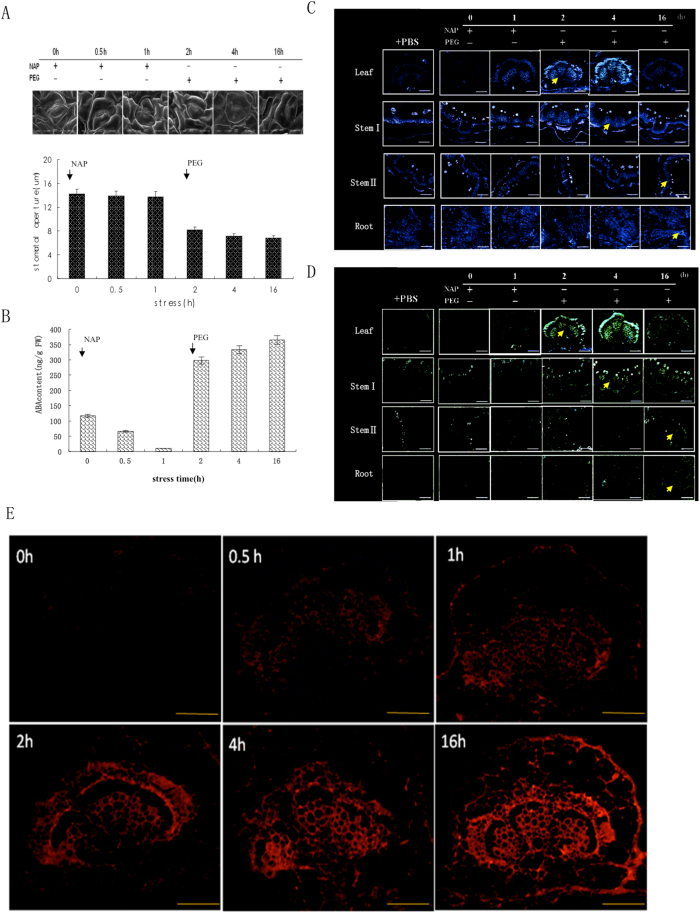
Effect of NAP on stomatal aperture, ABA level, and distribution of ABA and AhNCED1 in peanut in response to leaf stress. (**A**) Stomatal aperture in leaves on treatment of roots with NAP for 2 h, followed by PEG treatment from 2 h up to 16 h. (**B**) ABA levels in leaves following the same treatment regime described for A. Bars are means (±SD) of n = 3. (**C**) AhNCED1 immunostaining of leaf, stem and root following the same treatment regime described for A. Control samples were treated with PBS; blue fluorescence (arrows) indicates the presence of AhNCED1; no color indicates a lack of staining. (**D**) ABA immunostaining of leaf, stem and root following the same treatment regime described for A. Control samples were treated with PBS; green fluorescence (arrows) indicates the presence of ABA; no color indicates a lack of staining. Bars = 50 μm for leaf panels and 100 μm for stem and root panels. (**E**) ABA distribution at the subcellular level in root of peanut following leaf stress. Red fluorescence indicates positive immunostaining; no color indicates a lack of staining. Bars = 100 μm.

Treatment of leaves with Nap resulted in little change in stomatal aperture after 1 h (Fig. 5A), while at the same time point ABA content was markedly reduced (to 88% of values prior to treatment; Fig. 5B). Water stress was imposed 2 h after Nap treatment and, consequently, the ABA content in leaf tissue increased sharply to >50 times that of the level at 1 h. At the same time, mean stomatal aperture decreased rapidly to 64% of that 1 h after stress (Fig. 5A,B). During Nap treatment, AhNCED1 was present at very low levels in the peanut tissues tested. After PEG was imposed at 2 h, strong immunostaining signals were detected in the vascular tissue of leaves. However, AhNCED1 was not detected in the vascular tissue of stem Ι samples until the 4 h time point and not in the vascular tissue of stem II and taproot until 16 h (Fig. 5C). A similar pattern was observed for ABA (Fig. 5D). ABA localization at the subcellular level in leaves under leaf stress showed it to appear in the vascular cambium at the 0.5 h time point in this experiment. Subsequently, ABA-specific signals in the vascular cambium of leaf increased and also diffused to the xylem and epidermal cells (Fig. 5E).

### Different ABA transport in peanut depending on site of water stress

ABA transport was compared in peanut plants subjected to water stress at two different sites. We first applied labeled, exogenous ABA (^2^H_6_-ABA) to the top leaf of peanut seedlings for 4 h, then placed the taproot and top leaf in water or PEG solution for 2 h, respectively. As shown in Table 1, ^2^H_6_-ABA was detected in all three peanut tissues following treatment I, with a higher ^2^H_6_-ABA content in the top leaf than in stem and taproot. The ^2^H_6_-ABA content was increased in top leaf and decreased in stem when root was transferred from water to PEG solution (treatment II). However, when leaf was placed in PEG solution (treatment III), this effect was more pronounced, i.e. ^2^H_6_-ABA content was still higher in top leaf, and reduced to a greater extent than in treatment II in both stem and taproot (just 0.615 ng/g and 0.021 ng/g, respectively). We next applied ^2^H_6_-ABA to taproot for 4 h, then placed the root in water for 2 h (treatment IV) and assessed levels of ^2^H_6_-ABA in each of the three tissues. The ^2^H_6_-ABA content in all three organs was much higher than when the labelled exogenous ABA was applied to top leaf (treatments I–III), and was highest in taproot followed by stem and top leaf. Thus, ^2^H_6_-ABA content in taproot after treatment IV was 331.624 ng/g, which is over a hundred times that of treatment I. In treatment V, where root was transferred to PEG solution after the application of exogenous ABA to taproot, the ^2^H_6_-ABA content in top leaf increased from 68.609 ng/g to 272.121 ng/g, and decreased in taproot from 331.624 ng/g to 237.660 ng/g. Interestingly, when we placed the top leaf in PEG solution for 2 h after application of exogenous ABA to taproot (treatment VI), the ^2^H_6_-ABA content increased dramatically in top leaf, from 68.609 ng/g to 495.182 ng/g. At the same time, there was a substantial decrease in ^2^H_6_-ABA content in taproot, which was about 10 times lower than that in treatment IV (Table 1).

## Discussion

ABA synthesized in root tissues is thought to be transported to the apoplastic space of the leaf tissue by long distance transport, after which it enters the cytoplasm of leaf cells by simple diffusion without specific transporters^15^. Although 25–30% of the ABA in xylem sap might come from shoots due to recirculation of basipetally transported ABA in the phloem^16^, root ABA concentration increases in response to soil drying due to an increase in ABA biosynthesis by roots and ABA recirculation from shoots via phloem transport^17^. Therefore, understanding the relationship between ABA accumulation patterns at different stress sites and stomatal aperture is essential to predict long-distance ABA signalling responses to water stress. In a previous study, we found that biosynthesis of ABA in peanut plants subject to water deficiency may be dependent on developmental stage, with the roots being the initial site of ABA biosynthesis during the seedling stage, whereas during the fruiting stage ABA biosynthesis occurs initially in the leaf. The distribution patterns of ABA in seedling-stage peanuts in response to water stress are root-stem-leaf, while in fruiting-stage peanuts the distribution patterns of ABA are leaf-stem-root^12^. In this paper, we compared ABA biosynthesis and transport during water stress imposed at two different sites on the plant. We found that leaf stress resulted in a rapid increase in endogenous ABA content in leaves, 0.5 h after the stress treatment began. Immunostaining also showed that ABA, as well as AhNCED1, levels increased, and there was a concomitant, rapid reduction in mean stomatal aperture (Figs 1,2 and 4). However, when roots were under water stress, AhNCED1 and ABA immunostaining signals increased first in the root, i.e. at 1 h, with endogenous ABA content in leaf also increased at this time point, followed by the lower stem (2 h), the upper stem (4 h), and finally the leaves (16 h). In other words, the site where the stress is imposed in the peanut plant is where the initial response, in the form of ABA biosynthesis, takes places, as might be expected.

To confirm this, plants were treated with NAP, a potent ABA biosynthesis inhibitor that specifically targets NCED, for 2 h prior to the imposition of water stress. During NAP treatment, little or no AhNCED1 or ABA was detected in peanut tissues. After switching to PEG at 2 h, both AhNCED1 and ABA quickly appeared in the vascular cambium of root; immunstaining in other organs was relatively weak (Fig. 3). Yann^5^ found that ABA biosynthesized in Arabidopsis root cells was transferred to the apoplast, and then transported to the aboveground part of the plant through the xylem in the transpiration stream. In this way, ABA is able to regulate a series of related physiological activities (such as stomatal movement and photosynthesis) and to modulate gene expression in target tissues, thereby helping to repair injury resulting from stress^18^. ABA synthesised in leaves can be transferred to the roots through the phloem and part can be stored in the root, while the remainder can be recycled through xylem vessels^19^. In contrast, in plants pre-treated with NAP, PEG induced a rapid increase in AhNCED1 and ABA in the vascular tissue of leaf subjected to leaf stress. The ABA content in leaves increased rapidly (by 0.5 h), and the stomatal aperture declined (Figs 3 and 5). The distribution pattern of ABA in response to leaf stress was leaf-stem-root. Therefore, it seems clear that ABA biosynthesis and distribution depend on the site where water stress occurs in the peanut: leaf stress may lead to ABA synthesis and distribution initially in leaves, while root stress causes ABA to be synthesised and distributed first in the roots.

ABA and its metabolites are transported between compartments within a cell as well as between cells^20^,^21^,^22^. This raises the intriguing question as to which pool of ABA in a cell is perceived by the ABA receptors. It is possible that perception of ABA in different cellular compartments may exert different physiological outputs^23^,^24^,^25^. However, it remains unclear how ABA homeostasis is regulated and how the variable capacity for long distance ABA transport contributes to ABA signaling in crops subjected to water stress at different sites on the plant. We found that, when ^2^H_6_-ABA was applied to leaves, the ^2^H_6_-ABA content in the top leaf was higher than that in stem and taproot, and the ^2^H_6_-ABA content increased greatly markedly in the top leaf, and decreased both in stem and taproot, when either leaves or roots were placed in PEG solution after the application of ^2^H_6_-ABA. When ^2^H_6_-ABA was applied to taproot, the ^2^H_6_-ABA content in the taproot was higher than that in stem and top leaf, and ^2^H_6_-ABA levels in all three tissues were higher than in the experiments involving top leaf treatment. Interestingly, ^2^H_6_-ABA content also increased markedly in top leaf, and reduced both in stem and taproot, in treatments V and VI after application to taproot (Table 1). ABA is thought to be produced in most tissues, including leaves and roots. However, the expression of genes involved in ABA production at high levels in vascular parenchyma cells raises the intriguing possibility that ABA is transported from one tissue to another through the vascular system of plants^26^,^27^,^28^. As outlined above, we found that applied exogenous ABA is mainly transported from root to leaf and the absorbing abilities of exogenous ABA in different organs maybe effect ABA transportation and relatively drought resistance of peanut (Table 1). Wang *et al.*^29^ also found that exogenous ABA reducing membrane permeability and improving tolerance in specific leaf area of kiwifruit. But because we hadn’t measure total radiolabeled ABA contents in the treated plants so it is impossible to draw an appropriate conclusion about the absorbing ability. In a follow-up study, we plan to focus on analysis about absorbing abilities of exogenous ABA in peanut plants subjected to water stress at two different sites, based on complete data.

In this paper, we found that ABA biosynthesis, distribution, transport and the ability to induce stomatal closure were more pronounced in leaf than in root under conditions of leaf stress (Table 2). When peanut plants were subjected to root stress, ABA biosynthesis and distribution level were all more pronounced in root than in leaf. However, ABA transport and the ability to induce stomatal closure were still better in leaf than in root during root stress (Table 2). The above results show that, under root stress, ABA was rapidly synthesized in root, and accumulated largely in root vascular parenchyma, but then was quickly transported to the leaf tissues where it induced stomatal closure. When peanut plants were subjected to leaf stress, ABA biosynthesis initially increased in the leaf, then rapidly accumulated in the vascular cambium of leaves and induced stomatal closure; ABA produced in root tissues was also transported to the leaf tissues to maintain stomatal closure. The vascular system might be involved in the coordination and integration of this complex regulatory mechanism for ABA signal accumulation. These findings will help us to understand how ABA biosynthesis and distribution regulatory mechanisms lead to different physiological outputs in peanut subjected to water stress at different sites, and to advance a more complete understanding of the regulation of ABA transport.

## Methods

### Plant material

**Figure 6:**
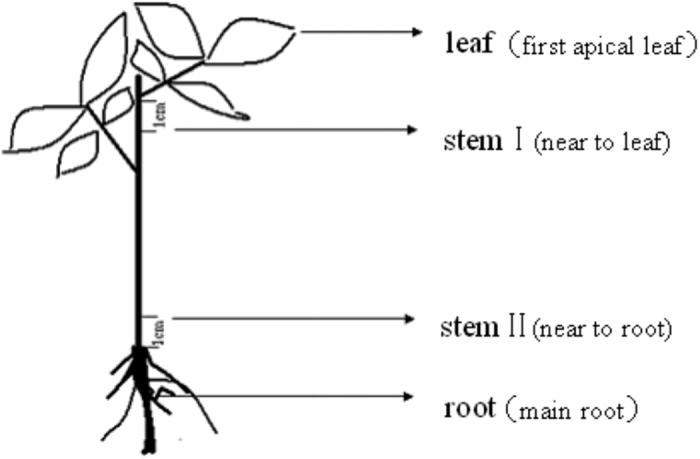


Peanut cultivar ‘Yueyou 7’, which is tolerant to drought stress, was provided by the Crop Research Institute, Guangdong Academy of Agricultural Sciences. Seeds were sterilized for 1 min in 70% ethanol and then rinsed 3–4 times with sterile deionized water. Seeds were planted in 2.5-liter soil-containing pots and grown to seedling (four-leaf) stage in a greenhouse (30 °C day, 20 °C night, with a 14 h light and 10 h dark cycle). The top leaf (first functional leaf) and taproot were transferred respectively onto filter paper containing 30% (w/v) polyethylene glycol (PEG) 6000 and treated for 1, 2, 4, 8, 16 or 24 h in the light. For naproxen treatment, plants were maintained in naproxen up to 2 h, and then PEG was replaced with naproxen (0.5 mM) after 2 h. For each time point, samples (0.5 g) of leaf (first functional leaf), stem Ι (near the first functional leaf, 1 cm), stem II (near the taproot, 1 cm), and root (taproot, 1 cm) were excised (Fig. 6). Aerial tissue was harvested at noon on the day specified. Excised samples were immediately frozen and stored at −80 °C until use. All treatments were performed in at least three independent experiments.

### Measurement of endogenous ABA content

Endogenous ABA was extracted from the frozen samples described above. Extraction in non-oxidative methanol: water (80:20, v/v), pre-purification through SepPak C18 cartridges (Waters, Milford, MA, USA) and HPLC fractionation using a Nucleosil C18 column (Macherey-Nagel, Germany) have been described previously^30^. The ELISA procedure was developed based upon the competition, for a limited amount of monoclonal anti-ABA antibody (1:2000 in 5% BSA/PBS; Invitrogen, CA, USA), between a standard ABA-BSA conjugate adsorbed onto the wells of a microtitration plate and free ABA extracted from the samples. Bound antibodies were labelled with a peroxidase-conjugated goat antibody raised against mouse immunoglobulins (Sigma-Aldrich, CA, USA), and peroxidase activity was then measured. A standard curve was established on each microtitration plate.

### Immunoenzyme localization assays

Samples (leaf, stem, root) from seedling-stage plants were embedded as described by Qin *et al.*^18^ and Hu *et al.*^14^ using a Technovit 7100 embedding kit (Heraeaus Kulzer, Wehrheim, Germany). Transverse sections (12 μm thick) were cut with a microtome (Sorvall MT-6000 ultramicrotome) and dried on glass slides. Immunoenzyme detection of AhNCED1 and ABA in the sections were performed using the streptavidin and biotinylated horseradish peroxidase complex (SABC) method. The sections were incubated in 3% H_2_O_2_ for 15 min at room temperature to block endogenous peroxidase activity. After three 5 min washes with distilled water, the sections were blocked with 10% BSA in PBS, then incubated with a dilution (10 mM PBS, 1% BSA, pH 7.2) of anti-ABA monoclonal antibodies (1:2000 in 5% BSA/PBS; Invitrogen, CA, USA) and anti-AhNCED1 antibodies (our laboratory storage, Hu *et al.*^14^) overnight at 4 °C. Slides were rinsed three times with PBS and incubated with a biotin-labeled goat anti-rat IgG antibody for 20 min at 37 °C. Following three washes with PBS, the sections were treated with SABC reagent (Sigma-Aldrich, CA, USA) for 20 min at 37 °C. After extensive washing in PBS supplemented with 0.02% (v/v) Tween 20 and PBS, the sections were stained with the AEC agent kit (Sangon, China) at room temperature. Sections were then washed with distilled water and immediately examined using an Olympus IX-70 microscope (Japan). Sections treated with PBS/BSA solution without primary antibody were used as negative control.

### Immunofluorescence localization assays

Cryofixation and freeze-substitution of leaf, stem and root were conducted as described by Kandasamy *et al.*^31^. In brief, the samples were rapidly frozen in liquid propane (−180 °C), freeze-substituted in acetone at −80 °C for 72 h, and then gradually brought to room temperature over an 8-h period. After rehydration through a graded acetone series, the seedling samples were washed in PME (50 mM PIPES, pH 7.0, 5 mM EGTA, 1 mM MgSO_4_, and 0.5% casein), permeabilized by treating with 1% Cellulysin (Calbiochem, Wehrheim, Germany) and 0.1% Pectolyase (Sigma-Aldrich) in PME containing protease inhibitors (complete mini EDTA-free protease inhibitor tablets; Sigma-Aldrich) for 20 min, washed in PME (5 min) and PBS (2 × 10 min), and squashed onto chromalum and gelatin-coated slides. Following air-drying, the leaf cells attached to the slides were further permeabilized in 0.5% Triton X-100 in PBS for 20 min and −20 °C methanol for 10 min. After rinsing in PBS, the slides were blocked for 1 h in TBST-BSA-GS (10 mM Tris-HCl, pH 7.5, 150 mM NaCl, 0.05% Tween 20, 2.5% BSA, and 10% goat serum) and then incubated in primary antibodies against AhNCED1 and ABA (5 μg·mL^−1^) in TBST-BSA-GS. After overnight incubation, the slides were rinsed with PBS, and then labeled for 3 h with 7-amino-4-methyl coumarin–conjugated anti-mouse secondary antibody (Sigma-Aldrich) for AhNCED1 and fluorescein FITC (IgA)–conjugated anti-mouse secondary antibody (Sigma-Aldrich) for ABA at 1:100 dilutions. The slides were then rinsed in PBS (3 × 10 min) and mounted with 80% glycerol in PBS containing 1 mg·mL^−1^ p-phenylenediamine (Sigma-Aldrich). The AhNCED1 and ABA were examined with a Leica confocal laser-scanning microscope (TCS-SP2).

### Measurement of stomatal aperture

A rectangular sample (1.0 × 0.5 cm) was cut from a peanut leaf and fixed in 4% glutaraldehyde for 4 h. The samples were rinsed three times with 0.1 M phosphate buffer and treated with 1% osmic acid for 3 h. The samples were washed with distilled water and dehydrated with a graded alcohol series and then stored in the freezer overnight. The dried leaf samples were mounted with conductive adhesive onto stubs, spray coated (6 milliamps, 4 min) in the ion sputtering apparatus (IB-5, EIKOIB, Japan), then observed with a scanning electron microscope (JSM-T300, JEOL, Japan). For each treatment, 25 leaf stomata were observed and photographed from three viewpoints, and pore width measured.

### Measurement of ^2^H_6_ -ABA

The incubation medium for binding assays contained 250 mM mannitol, 5 mM MgCl_2_, and 1 mM CaCl_2_ (except when determining the effects of Mg^2+^ and Ca^2+^ on ABA binding), 10 mM Tris/MES (pH 6.5, except when analyzing ABA binding at different pH), 70 nM ^2^H_6_-(±) ABA (TengLong, QingDao, China), except when analyzing ABA-binding kinetics where a step gradient of concentrations of ^2^H_6_-[±]ABA was used), and 30 ng of purified protein or the crude extract equivalent of 10 ng of protein. The total incubation volume of each assay was 200 μL. The mixtures were incubated at 4 °C for 1 h and then quickly placed on ice. After the addition of 50 μL of 0.5% (w/v) DCC (dicyclohexyl carbodiimide) to remove free ^2^H_6_-ABA by adsorption, the mixtures were maintained on ice for 10 min and then centrifuged to remove DCC, and then measured by ICP-MS (ELAN6000, AGILENT, Japan). The specific binding was determined by the difference between the radioactivity bound to the purified proteins or crude extract incubated only with ^2^H_6_-ABA (total binding) and the radioactivity bound in the presence of 1,000-fold molar excess of unlabeled (±) ABA (Sigma-Aldrich; non-specific binding). The unlabeled (±) ABA was added into the incubation medium at the same time as the ^2^H_6_-ABA.

## Additional Information

**How to cite this article**: Hu, B. *et al.* The site of water stress governs the pattern of ABA synthesis and transport in peanut. *Sci. Rep.* **6**, 32143; doi: 10.1038/srep32143 (2016).

## Figures and Tables

**Table 1:** Exogenous ABA leaf (^2^H_6_-ABA) content in tissues of peanut subjected to water stress at two different sites.

Marking methods	Measured value of treated position/^2^H_6_-ABA (ng/g)		
	Top leaf	Stem	Taproot
I (Marking at top leaf for 4 h, Then put root into water for 2 h)	16.739 ± 0.012	5.455 ± 0.009	2.798 ± 0.010
II (Marking at top leaf for 4 h, Then put root into PEG for 2 h)	20.210 ± 0.014	2.613 ± 0.008	2.046 ± 0.006
III (Marking at top leaf for 4 h, Then put leaf into PEG for 2 h)	25.080 ± 0.015	0.615 ± 0.007	0.021 ± 0.004
IV (Marking at taproot for 4 h, Then put root into water for 2 h)	68.609 ± 0.020	279.987 ± 0.032	331.624 ± 0.028
V (Marking at taproot for 4 h, Then put root into PEG for 2 h)	272.121 ± 0.029	212.154 ± 0.035	237.660 ± 0.026
VI (Marking at taproot for 4 h, Then put leaf into PEG for 2 h)	495.182 ± 0.041	128.456 ± 0.025	25.638 ± 0.019
Control	0.000	0.000	0.000

**Table 2:** Analysis with correlation capabilities of ABA in peanut leaf and root under different-sources water stress.

	Leaf-sources water stress		Root-sources water stress	
	Leaf	Root	Leaf	Root
ABA biosynthesis ability	+++	+	++	+++
ABA distribution level	+++	+	++	+++
ABA transport ability	++++	+++	+++	++
Induce stoma close ability	++++	++	+++	+

“+’’ weak ability; “++” normal ability; “+++” strong ability; “++++” very strong ability.
